# Radionuclide and Trace Element Distribution in Grey Wolves (*Canis lupus*): Implications for Environmental Contamination and Transfer in Terrestrial Ecosystems

**DOI:** 10.3390/toxics14050425

**Published:** 2026-05-12

**Authors:** Maja Lazarus, Božena Skoko, Mikael Hult, Tatjana Orct, Maja Ferenčaković, Ivana Coha, Josip Kusak, Slaven Reljić, Gerd Marissens, Heiko Stroh

**Affiliations:** 1 Division of Occupational and Environmental Health, Institute for Medical Research and Occupational Health, 10000 Zagreb, Croatia; torct@imi.hr; 2 Division for Marine and Environmental Research, Ruđer Bošković Institute, 10000 Zagreb, Croatia; bozena.skoko@gmail.com (B.S.); ivana.coha@irb.hr (I.C.); 3 European Commission, Joint Research Centre (JRC), 2440 Geel, Belgium; mikael.hult@ec.europa.eu (M.H.); gerd.marissens@ec.europa.eu (G.M.); heiko.stroh@ec.europa.eu (H.S.); 4 Faculty of Agriculture, University of Zagreb, 10000 Zagreb, Croatia; mferencakovic@agr.hr; 5 Faculty of Veterinary Medicine, University of Zagreb, 10000 Zagreb, Croatia; kusak@vef.hr (J.K.); slaven.reljic@gmail.com (S.R.); 6 College of Sciences, Koç University, 34450 Istanbul, Türkiye; 7 School of Biological Sciences, University of Utah, Salt Lake City, UT 84112, USA

**Keywords:** radiocaesium, radiolead, non-essential element, transfer parameter, concentration ratio, wildlife, inorganic pollutants, bone, soft tissue

## Abstract

Anthropogenic pollution may impose additional pressure on European populations of large protected carnivores due to the systemic toxicity of contaminants such as cadmium, lead, and radiocaesium (^137^Cs). Our aim was to carry out ^137^Cs, radiopotassium (^40^K), and stable element distribution analysis through seven tissues of grey wolves (*Canis lupus*) from temperate forests of Croatia using ultra-low background gamma-ray spectrometry and ICPMS, respectively. In addition, radiolead (^210^Pb) massic activity was quantified in femoral bone. The massic activity of ^137^Cs in the heart, kidney, liver, spleen, lungs, and femoral bone (in decreasing order) ranged from 9–61% relative to muscle and showed strong inter-tissue correlations. However, correlations between radionuclides and their stable analogues in wolf tissues indicated considerable uncertainty in the use of stable element data for radiological risk assessment. In addition, concentration ratios (CR_whole organism-soil_) derived from stable element data should be applied with caution when radionuclide data are lacking. Overall, radionuclide activities and element levels not subject to homeostatic regulation in grey wolves were comparable to or lower than those reported for other populations, particularly those from sub-Arctic regions. Despite being apex terrestrial predators, wolves inhabiting temperate ecosystems do not currently appear to be at risk of adverse health effects from exposure to the most relevant inorganic anthropogenic pollutants.

## 1. Introduction

Human activities generate pollutants that affect both abiotic environmental compartments and the health of biota worldwide. Radiocaesium (^137^Cs) is a toxicologically important anthropogenic gamma-ray-emitting radionuclide whose environmental fallout typically contributes less than 1% to the total annual radiation dose, except following nuclear power plant accidents such as Chernobyl (1986) and Fukushima (2011). With a physical half-life of 30.0 years [1], the continued monitoring of ^137^Cs in biota is essential for long-term radiological risk assessment [2]. Due to ionic mimicry with potassium (K), radiocaesium preferentially accumulates in the muscle tissue of humans and animals, making muscle ^137^Cs a sensitive marker of recent dietary exposure. In contrast, radionuclides that mimic calcium (Ca), such as strontium (^90^Sr) and lead (^210^Pb), preferentially accumulate in bone, where ^210^Pb reflects long-term exposure. Radiolead is a highly radiotoxic beta- and gamma-emitting radionuclide with a half-life of 22.2 years [3] and occurs naturally as part of the uranium decay series, with environmental levels further enhanced by anthropogenic activities, including uranium and phosphate mining, fossil fuel combustion, and fertilizer production [4]. In line with radionuclides, biota is concurrently exposed to various non-essential stable elements (e.g., arsenic, As; cadmium, Cd; mercury, Hg; lead, Pb) as priority pollutants [5], whose toxicity in mammals captures almost all organs and systems and impairs the homeostasis of essential elements [6,7,8,9,10].

The expansion of human-centered focus to include environmental protection has increased the need for data on various pollutant occurrences and biota-to-wildlife transfers in terrestrial ecosystems [11,12]. Forest ecosystems exhibit a higher uptake, biological recycling and storage of radionuclides and chemical pollutants than agricultural or aquatic ecosystems [2,13,14], making their monitoring particularly relevant for the protection of forest biota and humans consuming wildlife or forest products. Within a One Health framework, assessing dose rates and biological effects of persistent trace elements and long-lived anthropogenic radionuclides in wildlife is a key priority [15]. In Europe, increasing emphasis has been placed on the conservation of large carnivores [16] and on understanding pollutant-related adverse effects on their health, including reproduction, body condition, and immune function [17]. As apex predators and umbrella species, large carnivores play key ecological roles and, through the progressive accumulation of contaminants along the terrestrial food web, serve as valuable sentinel species [17,18,19].

Grey wolf (*Canis lupus*) as an apex carnivore was previously employed in trace element [20,21,22,23,24,25,26,27], radionuclide [28,29,30], and persistent organic pollutant [31] monitoring studies due to its high trophic position shared with humans; comparable mechanisms of contaminant uptake, distribution, metabolism, and excretion; relative longevity; and wide geographic distribution across the globe [32]. Despite listed attributes, between-tissue data on radionuclide and stable element concentrations in wolves remain non-existent, particularly outside Arctic populations characterized by *Rangifer* spp.-based diets and elevated radionuclide levels [30]. Although organ-specific pollutant distribution is critical for assessing radiosensitivity and stable element toxicity, concentrations in tissues other than primary accumulation sites have been sparsely studied [19,30,33,34]. Obvious obstacles in radiochemistry were small sample tissue masses and limited detector sensitivity under aboveground gamma-ray spectrometry conditions. The underground laboratory HADES [35] provides ultra-low background conditions that enable high-sensitivity radionuclide detection in biological samples [36,37].

To estimate internal exposure in large carnivorous mammals, radiological protection authorities recommend quantifying radionuclide transfer from soil to animals using concentration ratios (CRs) [38,39,40]. The background for such methodology comes from the good correlation of wildlife muscle radiocaesium massic activities with soil deposition at capture locations [41,42]. Contaminant transfer to top predators like wolves was shown to be highly species-specific [18,41] and thus highlighted a challenge when using generic CRs for carnivorous mammals category in radiological risk assessment [39]. A clear data gap for wolves from forest ecosystems of temperate climate is also obvious in the online wildlife transfer database [12]. When radiological safety assessments lack massic activity data or CRs for a given radionuclide–species combination, the use of stable multielement data or surrogate information from similar species has been recommended [43]. However, this approach may lead to an underestimation of risk, as several studies have reported mismatches between radionuclide massic activities in animal tissues and CR values, and those of their stable element analogues ([39,44,45] and references therein). In addition, ionic competition has been shown to affect both stable element concentrations [7,8,9,10] and radionuclide uptake (e.g., ^137^Cs and ^40^K [46]), yet mutual interactions between stable and radioactive elements in animal tissues remain poorly described in the literature [44,45].

In Southeast Europe, brown bears (*Ursus arctos*) and grey wolves exhibited higher ^137^Cs massic activities than sympatric herbivores and omnivorous golden jackals (*Canis aureus*), but levels comparable to omnivorous wild boar (*Sus scrofa* [28,47]). In contrast, during the early post-accident period in the Fukushima Daiichi Nuclear Power Station, wild boar in Japan showed substantially higher ^137^Cs massic activities than Asian black bears (*Ursus thibetanus*), highlighting context-dependent trophic transfer dynamics [41].

According to the IUCN Red List, the grey wolf is classified globally as a species of Least Concern [48], while European Commission (Directive (EU) 2025/1237) recently downgraded the legal status of species to protected (under Habitats Directive and Bern Convention). The vulnerability of European wolf populations, driven by habitat fragmentation, illegal killing and other human/illness pressures [49], may be further exacerbated by pollutant-related health effects [17]; thus, regular monitoring is important.

Our aim was to study distribution of environmentally relevant radionuclides and stable elements among seven tissues (muscle, heart, lungs, spleen, liver, kidney, and femoral bone) of the European grey wolf inhabiting the temperate forests of Croatia. In addition, we assessed the transfer of pollutants from soil to wolf by using the concentration ratios to acknowledge the existing data gap on this protected species and improve conservation-centered stable element and radiological risk assessment. Also, we estimated the possibility of using transfer parameters derived from stable elements as analogues for radionuclide transfer in large terrestrial carnivores.

## 2. Materials and Methods

### 2.1. Study Area and Sampling

Ten grey wolf carcasses (Table 1) were collected between 2014 and 2018 by the Intervention Team for Wolves and Lynx (approved by the Ministry of Environmental Protection and Green Transition) from three regions of Croatia: North (Gorski Kotar and Banovina), Central (Lika), and South (Dalmatia).

Sampling sites encompassed areas of permanent presence of the Dinaric–Balkan wolf population (approx. 43 packs in Croatia), while the mortality of one individual (W5) was recorded in the Banovina region, where wolves occur only occasionally [49]. The previously established total home range mean of two packs from the southern region was 150.5 km^2^ (100% MCP; [50]. Despite variation related to sex and body condition, wolves are typically regarded as adults from the age of two years [51]. Distinct climatic zones in the studied area (continental temperate, mountainous, and Mediterranean), geomorphological features [52], and human land-use practices, particularly livestock breeding, result in regional differences in prey availability. In the northern region, wolf diet is dominated by wild prey, primarily wild boar, red deer (*Cervus elaphus*), and roe deer (*Capreolus capreolus*), whereas, in the central and southern regions, wolves more frequently prey on domestic animals, including goats, sheep, horses, cattle, and dogs, reflecting the greater prevalence of extensive livestock farming [53,54].

**Table 1:** The biometric and sampling data for ten grey wolves (*Canis lupus*) sampled during the 2014–2018 period in Croatia ^a^.

Wolf ID	Analyte ^a^	Sampling Date	Sampling Latitude (*n*)	Location Longitude (E)	Sampling Area	Age (y)	Sex	Body Mass (kg)	Body Length (cm) ^b^	Body Condition Index ^c^
W1	SE	12 March 2014	43.62786	16.70859	South	2	female	24.7	114	−4
W2	SE, ^137^Cs, ^40^K, ^210^Pb	13 March 2014	45.336203	14.591506	North	0.9	female	/	/	/
W3	SE	20 December 2014	44.113574	16.196741	South	0.8	male	18.5	118	−12
W4	SE, ^137^Cs, ^40^K, ^210^Pb	29 October 2015	44.80317	15.562267	Central	0.5	female	22	102	−2
W5	SE, ^137^Cs, ^40^K, ^210^Pb	18 November 2015	45.399517	16.045754	North	1.5	male	30	135	−8
W6	SE, ^137^Cs, ^40^K	22 February 2017	43.601273	16.198155	South	1.5	female	24.3	120	−7
W7	SE, ^137^Cs, ^40^K, ^210^Pb	17 May 2017	44.669912	15.722203	Central	2	female	36.4	120	5
W8	SE, ^137^Cs, ^40^K	13 June 2017	44.268827	15.966538	Central	2	male	41.4	126	7
W9	SE, ^137^Cs, ^40^K, ^210^Pb	7 July 2017	45.378639	14.514897	North	2.4	male	37.4	124	4
W10	SE	25 February 2018	44.357989	15.695208	Central	2.5	male	34.1	120	3

^a^ SE—stable elements in all tissues (muscle, heart, spleen, lungs, liver, kidney, femoral bone); ^137^Cs and ^40^K in soft tissues, ^210^Pb in femoral bone. ^b^ Measured from the tip of the nose until the sacrococcygeal joint (body length without tail). ^c^ Body condition was calculated from residuals of an ordinary least squares regression of body length and body mass [55,56] of 196 wolves residing in Croatia in the period 1997–2025 (J. Kusak, 2026, Personal Communication).

Since 1995, the grey wolf has been a strictly protected species in Croatia, with conservation measures aligned with the Bern Convention and the EU Habitats Directive but, recently, with Directive (EU) 2025/1237, its status changed to protected species. The study regions are characterized by the Dinaric Mountains (up to 1830 m a.s.l.) and carbonate bedrock hills largely covered by dense forests dominated by beech (*Fagus sylvatica*), fir (*Abies alba*), and Norway spruce (*Picea abies*), particularly in the northern and central regions [57]. Soil concentrations of toxicologically relevant stable trace elements (Cd, Hg, and Pb) in the study areas exceed national median values primarily due to natural geochemical background, while additional anthropogenic inputs from nearby urban centers and industrial activities contribute to elevated levels of a broader range of trace elements [52]. Radiocaesium massic activities in soils of the study area are higher than the Croatian average, largely reflecting fallout from the Chernobyl accident, with the highest levels reported in the central region [46,58]. In contrast, soil activities of ^40^K and ^210^Pb are comparable to national background levels [46].

The causes of death for two wolves (W1 and W3; Table 1) were illegal killing, whereas the remaining eight individuals were road casualties. Morphological characteristics, sex, and location were recorded on site. Body length was measured from the tip of the nose to the last vertebral bone (excluding the tail). Body condition index (BCI) was calculated from residuals of an ordinary least squares regression of body length and body mass [55,56] of 196 wolves residing in Croatia and measured in the period 1997–2025 (J. Kusak, 2026, Personal Communication). Age was estimated based on body size, tooth wear [59], and the date of death. Skeletal muscle, heart, spleen, lung, liver, kidney tissue and femoral bone were dissected at the Faculty of Veterinary Medicine Zagreb and stored in plastic bags at −20 °C until chemical analysis. Residual connective tissue was scraped off the femoral bone, and a 0.2 g subsample from the mid-diaphysis was cut using a diamond-coated blade mounted on a Dremel^®^ 4000 rotary tool for stable element analysis, as described previously [60]. All tissues were weighed before and after freeze-drying for 72 h (HyperCOOL HC3055, LabTech Srl, Korea) to determine moisture content.

### 2.2. Radionuclide Massic Activity Quantification

Dried soft tissues of seven wolves were homogenized in a stainless steel blender. Five femoral bones were crushed into small pieces after immersion into liquid nitrogen and pulverized by cryogenic grinding (FreezerMill 6875D; SPEX Sample Prep, Metuchen, NJ, USA). Around 3–5 g of pulverized soft tissues and 60–100 g of bone powder were packed in cylindrical Teflon containers (7 cm^3^ and 76 cm^3^, respectively) for gamma-ray spectrometry. The massic activity of the samples was determined in the research facility HADES located 225 m below ground [35], on two HPGe-detectors: ^137^Cs and ^40^K in soft tissues on a detector Ge14 (SAGe-well detector, Mirion Technologies, San Ramon, CA, USA), and ^137^Cs, ^40^K and ^210^Pb in bone samples on a detector Ge4 (XtRa-detector, Mirion Technologies, San Ramon, CA, USA). The typical measurement time was 7 days (but ranged from 5 to 26 days for practical reasons). For the calculation of ^137^Cs, ^40^K and ^210^Pb massic activities, peaks at 661.655(3) keV, 1460.822(6) keV, and 46.539(1) keV were used, respectively. Full-energy peak efficiencies were determined using the efficiency transfer method [61] by first establishing, for each detector and geometry, an efficiency curve using a multi-nuclide calibration source. Then, corrections for geometry and composition were calculated using Monte Carlo simulations. The efficiency transfer factors were calculated using Monte Carlo simulations with the HPGe3-module of the EGSnrc-code [62]. The massic activity of each radionuclide was calculated using the Excel-based analysis software GLysis V2022.05.11 [62], taking into account emission probabilities and half-lives from the Decay Data Evaluation Project [3]. The minimum detectable activities (decision thresholds with α = 0.05) were typically 51 mBq/kg, 550 mBq/kg and 540 mBq/kg for ^137^Cs, ^40^K, and ^210^Pb, respectively, in a 4.08 g sample of muscle tissue measured for 7 days. All massic activities were decay-corrected to the date of animal sampling using their physical half-life [1,3].

To obtain robust values and realistic uncertainties, a number of metrological quality control measures were performed: a sensitivity analysis for optimal detector/sample configuration, an investigation of detector bias based on proficiency tests, and an investigation of the impact of changes of filling height in the sample containers [63]. Uncertainty budget analysis for ^137^Cs in muscle resulted in a relative uncertainty of 4.4%, with the two dominating uncertainty contributions being counting statistics, 2.5%, and efficiency, 3%.

### 2.3. Stable Element Quantification

Dry tissue subsamples (0.2–0.3 g) of ten wolves were acid digested using an UltraCLAVE IV (Milestone, Sorisole, Italy) high-pressure microwave digestion system prior to stable element (As, Ba, Ca, Cd, Co, Cs, Cu, Fe, Hg, K, Mn, Mo, Pb, Se, Sr, Tl, U, V, Zn) quantification by inductively coupled plasma mass spectrometry (ICPMS; Agilent 8900, Agilent Technologies, Santa Clara, CA, USA), following a previously described method [64]. Purified nitric acid (p.a. 65%, Merck, Darmstadt, Germany; SubPUR/DuoPUR, Milestone, Italy) and ultrapure water (Smart2Pure 6 UV/UF, Thermo Scientific, Waltham, MA, USA, SAD) were used for sample preparation and dilution. Matrix-matched animal tissue certified reference materials (ERM-BB184 Bovine muscle, BCR-185R Bovine liver and BCR-186 Pig kidney; Institute for Reference Materials and Measurements, Geel, Belgium, and 1577a Bovine liver; National Institute of Standards and Technology, Gaithersburg, MD, USA) were processed and analyzed alongside samples for quality control. Element recoveries ranged from 93 to 110%. All stable element levels are expressed on a dry mass basis.

### 2.4. Data Analysis

Radionuclide and stable element concentrations were summarized using arithmetic mean ± standard deviation, geometric mean, median, and range. Given the small sample size (*n* = 5–7 for radionuclides, *n* = 10 for stable elements), non-parametric and permutation-based methods were used throughout the statistical analysis. Associations between radionuclide massic activity and their stable element analogue levels (^137^Cs and Cs, ^40^K and K in all tissues; ^210^Pb and Pb in femoral bone) were assessed using Bayesian estimation with a bivariate Student-t likelihood, following the robust correlation model of Kruschke [65]. One individual (W4, Table 1) acted as a biologically plausible outlier and was retained in all analyses. The Student-t likelihood accommodates outlying observations by estimating heavier tails than the normal distribution, thereby down-weighting extreme data points without excluding them. Weakly informative priors were specified as follows: *ρ* ~ Uniform(−1, 1) for the correlation coefficient, *ν* − 1 ~ Exponential(1/29) for the degrees of freedom, location parameters centered on the corresponding sample means with scale 2.5 × SD, and scale parameters *σ* ~ HalfNormal(2.5 × SD). For each tissue, the posterior distribution of the correlation coefficient ρ was sampled using Markov chain Monte Carlo (4 chains × 2000 draws, 1000 tuning steps; PyMC v5; [66]) and summarized as the posterior mean with 95% highest density interval (HDI). Model convergence was verified by $\hat{R}$ < 1.01 and effective sample size >400 for all parameters. This approach was chosen over classical correlation tests because, with *n* = 5–7, frequentist *p*-values lack sufficient statistical power, while the Bayesian HDI provides a direct and interpretable measure of uncertainty in the estimated association. Differences between relative tissue distributions of ^137^Cs and stable Cs were assessed using two-sided paired permutation tests (10,000 sign-flip permutations) within each tissue. Inter-tissue correlations for ^137^Cs, Cd, and Pb were calculated using Spearman rank correlation and presented as heatmaps for qualitative pattern description, without formal significance testing, given the exploratory nature and small sample size. The influence of sex, age, and BCI on contaminant levels was tested in target organs of the most relevant anthropogenic pollutants (^137^Cs and Cs in muscle, Cd in kidney, Pb in femoral bone), using permutation correlation tests for continuous predictors (age, BCI) and permutation tests for difference in means for sex comparison (10,000 permutations each). The data were analyzed using JMP Pro 19 (JMP Statistical Discovery LLC, Cary, NC, USA). Visualizations of results were performed using TIBCO Statistica^®^ software, version 14.0.0.15 (TIBCO Software Inc., Palo Alto, CA, USA) and SAS 9.4 software (SAS Institute Inc., Cary, NC, USA).

We calculated concentration ratios (CR_wo-soil_) as a simplistic approximation of transfer of radionuclides (^137^Cs and ^210^Pb) and stable elements (Ca, Cd, Cu, Cs, Mn, Pb, and Zn) between soil and wolves inhabiting the Croatian forest ecosystem, as previously suggested [38,40,43], according to Equation (1):

(1) $${CR}_{wo-soil}=\;\frac{\begin{array}{c} c_{wo}(\frac{Bq}{kg}\mathrm{or}\frac{\mu g}{kg}\mathrm{wet}\;\mathrm{mass}) \end{array}}{\begin{array}{c} c_{soil}(\frac{Bq}{kg}\mathrm{or}\frac{\mu g}{kg}\mathrm{dry}\;\mathrm{mass}) \end{array}}$$

The nominator (c_wo_) represents radionuclide massic activity or stable element level in the whole wolf’s body and a denominator (c_soil_) represents respective massic activity or stable element level in the soil of study area. CRs were calculated only for stable elements that had available soil level data. Muscle ^137^Cs activity, Ca, Cd, and Cu, bone Mn, and hepatic Zn level quantified in this study were assumed to represent the whole organism [38,43]. The conversion factor (f = 0.16) for Pb bone to whole organism was taken from the IAEA handbook on wildlife transfer parameters [38], taking into account the 0.8 ratio of dry bone mass to wet mass. To recalculate concentrations in soft tissues from dry to wet mass, the moisture content of muscles (75%), i.e., corresponding conversion factor (f = 0.25, [38]) was multiplied, with concentration expressed on a dry mass. Moisture content determined for the heart, spleen and kidney of wolves was 76% for heart (f = 0.24), 77% for lungs (f = 0.23), and 70% for liver (f = 0.3). The massic activity of ^137^Cs in the soil of the studied area was calculated as the arithmetic mean gained from values reported by Šprem et al. (109 Bq/kg [28]) and Babić et al. (141 Bq/kg [46]) and was decay-corrected to the date of wolves’ sampling before being included in our calculations. The massic activity of ^210^Pb in soil was adopted from Babić et al. [46]. Stable soil Ca, Cd, Cu, Mn, Pb, and Zn concentrations were adopted from a geochemical atlas [52].

Toxicological risk assessment regarding stable elements was obtained according to thresholds available in the literature.

## 3. Results

### 3.1. Influence of Age, Sex and Body Condition

Because several variables, particularly non-essential elements and ^137^Cs, showed skewed distributions, median, geometric mean, and range are reported, in addition to arithmetic mean values in Table 2 and Table A1.

Ultra-low background conditions in the underground laboratory enabled the quantification of ^137^Cs massic activities in all wolf samples and tissues. As expected, the highest ^137^Cs activities were observed in muscles (Table 2), reaching 193 Bq/kg dry mass (dm) in the youngest sampled individual (W4; Table 1). This 0.5-year-old female had a below-average body condition and showed outlier values of ^137^Cs and stable Cs in all tissues, whereas bone ^210^Pb and other stable non-essential elements remained within the range observed in other wolves.

Stable element levels were quantified in most of the seven analyzed tissues, with a few exceptions where values were below the detection limit: Hg (10%), Pb (20%), and U (80%) in muscle; U (80%) in heart; As (10%) and Hg (20%) in lungs; and Cd (30%), Fe (80%), and Mo (100%) in bone (Table 2; Table A1).

The assessment of age as a biological factor potentially influencing ^137^Cs massic activity in wolves showed no significant association (r = −0.60, *p* = 0.18). Radiocaesium and Cs were similar between the sexes, with only non-significant tendencies toward higher ^137^Cs (∆mean = −56.0, *p* = 0.39) and Cs values (∆mean = −516.0, *p* = 0.60) in females. No significant influence of body condition index on ^137^Cs activity was detected in wolves in the present study (r = −0.004, *p* = 0.99). Regarding stable elements, the effect of biological factors was tested on the most toxicologically relevant anthropogenic pollutants in their target tissues, namely, Cd in kidney (age: r = 0.045, *p* = 0.91, sex: ∆mean = −358, *p* = 0.77, BCI: r = 0.13, *p* = 0.77), and Pb in femoral bone (age: r = 0.19, *p* = 0.60, sex: ∆mean = 214, *p* = 0.80, BCI: r = −0.16, *p* = 0.69), and showed no significant influence in wolves.

### 3.2. Distribution Among Tissues

Radiocaesium and ^40^K distribution differed among wolf tissues, while ^210^Pb was detectable only in femoral bone (Table 2).

**Figure 1:**
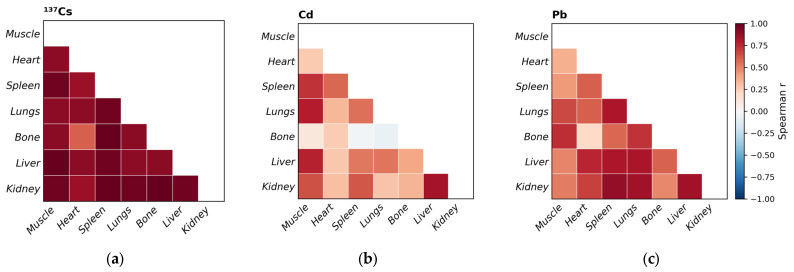
Heatmap of Spearman correlation coefficients (r) for ^137^Cs massic activity, *n* = 7 (**a**), Cd (**b**), and Pb levels, *n* = 10 (**c**), between seven tissues of grey wolves (*Canis lupus*) sampled during the 2014–2018 period in Croatia.

Tissues such as muscle, heart, spleen, and lungs showed lower accumulation of stable elements without known biological function (i.e., non-essential elements) in wolves compared to the liver and kidney (Table 2; Table A1). In contrast, bone acted as a target tissue for As, Ba, Pb and Sr. Arsenic can accumulate through substitution for phosphate ions, whereas Ba, Pb, and Sr are incorporated into bone by replacing Ca ions. Owing to the chemical similarity between Cs and K ions, muscle served as the primary tissue for Cs distribution in wolves in this study. Consistent with earlier reports describing the strong affinity of Cd and Hg for wolf kidneys [22,24,26,27,67,68], our results also identified the kidney as a major accumulation organ for Tl and U. Inter-tissue correlations (Figure 1b,c) further illustrate the relationships in the distribution of the most toxicologically relevant elements among organs, with the strongest significant correlations for both Cd and Pb observed between the kidney and liver (r = 0.83 and r = 0.84, respectively). Strong associations were consistently observed between ^137^Cs massic activity in muscle and that in other tissues of wolves (Figure 1a).

### 3.3. Radionuclide vs. Stable Element Analogue

**Figure 2:**
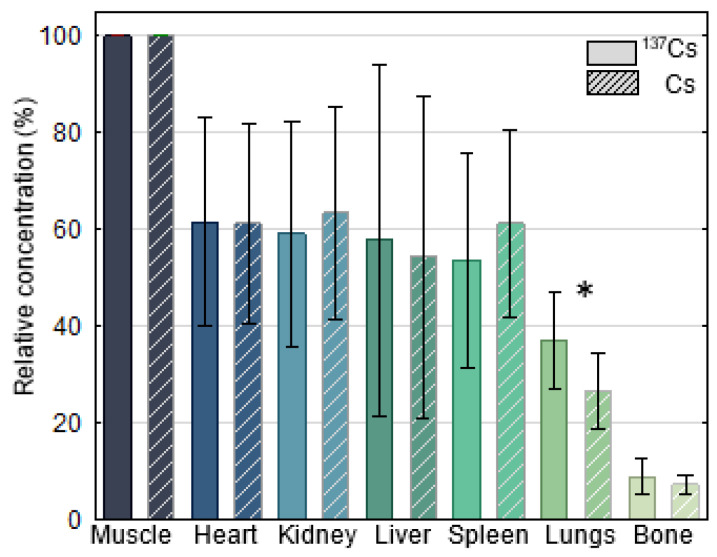
Relative to muscle, the distribution of radiocaesium (^137^Cs, solid column) and stable caesium (Cs, striped column) in the organs of grey wolves (*Canis lupus*) sampled in Croatia during the 2014–2018 period. Statistically significant differences between relative ^137^Cs and Cs within individual tissues are indicated by asterisk.

The relative massic activity of ^137^Cs and Cs in the heart, kidney, liver, spleen, lungs, and femoral bone (listed in decreasing order) ranged from 7 to 66% of those in muscle (Figure 2). The distribution patterns of relative ^137^Cs resembled that of relative Cs in wolves, and were consistent with previous reports [69], indicating that ^137^Cs activity in tissues had largely reached equilibrium. However, a significant deviation between relative ^137^Cs and relative stable Cs was observed in the lungs (∆mean = 10.9, *p* = 0.016), which, owing to their high blood content, may be particularly prone to variability.

Correlation analysis revealed strong associations between ^137^Cs, ^210^Pb, and their stable analogues across wolf tissues (Table 3), although these estimates were accompanied by considerable uncertainty, as indicated by the wide density intervals. This uncertainty likely reflects both the high massic activities observed in wolf W4 relative to the other individuals and the small sample size (*n* = 5–7). Therefore, under these conditions, stable element concentrations cannot be considered reliable proxies for radiological risk assessment. This approach has been suggested in situations where radionuclide activity data are unavailable, a common constraint in studies of large terrestrial carnivores [39].

### 3.4. Concentration Ratios

Table 4 summarizes soil-to-wolf concentration ratios (CRs) for stable elements (Ca, Cd, Cu, Mn, Pb, and Zn), as well as for ^137^Cs and ^210^Pb. CR values derived from ^210^Pb and stable Pb differ by approximately one order of magnitude. The calculated transfer parameters are further compared with literature values for the respective elements across diverse mammalian categories.

### 3.5. Toxicological Relevance of Stable Elements

According to Table 5, none of the measured non-essential elements in Croatian wolves exceeded levels associated with toxic effects in terrestrial mammals [71,72,73,74] or in domestic dogs [75]. In addition, adequate or high reference levels of essential elements in domestic dogs [75] were compared to the one quantified in grey wolves from this study.

## 4. Discussion

### 4.1. Radionuclides

#### 4.1.1. Influence of Age, Sex and Body Condition

Pronounced inter-individual variability in radiocaesium massic activities has previously been reported in large mammals, even within the same study area, reflecting biological factors that vary seasonally [18,19,41,77,78]. In addition to local geochemistry, which may explain up to 80% of muscle ^137^Cs variability [79], diet composition, seasonal resource use, the bioavailability of dietary elements, and species-specific physiological and life-history traits can further influence contaminant transfer to top predators. Muscle ^137^Cs and stable Cs, markers of short-term dietary exposure [42], were markedly higher than ^210^Pb and non-essential elements accumulating in slow-turnover tissues (e.g., bone, liver, kidney), when comparing W4 with other wolves from this study. This individual originated from the central study area (Lika region), where the highest soil ^137^Cs activities in Croatia have been reported [46,58]. Increased soil intake may have occurred through intentional ingestion associated with mineral deficiency, digging, or fur licking [80], behaviors that may be more pronounced in young animals [81]. In addition, young age is associated with physiological traits such as higher food intake relative to body weight, increased gastrointestinal absorption and metabolic rate, lower excretion rates, and potential essential element deficiencies, all of which can enhance the exposure, uptake, and accumulation of non-essential elements [82,83].

The irrelevance of age for ^137^Cs massic activities found in this study is in agreement with earlier findings for Swedish bears, but contrasts with reports for lynx (*Lynx lynx*), in which adults exhibited higher massic activities [79,84]. Differences in prey selection and physiological characteristics have been suggested as possible explanations for age-related variation in lynx [84]. Likewise, although males and females may differ in home-range size during certain breeding-related life-history periods [85], the lack of significant sex differences in ^137^Cs activities in Croatian grey wolves is consistent with observations reported for bears and lynx [79,84]. To the best of our knowledge, the effect of body condition on ^137^Cs activity has not previously been evaluated in large mammals. Nevertheless, these conclusions should be interpreted with caution because of the small sample size.

In general, ^137^Cs massic activities in grey wolf muscles in this study were relatively low (1.15–193 Bq/kg dm; Table 2) and lower than those reported in Sweden (70–8410 Bq/kg dm, 2010–2011 period [29]). Mean bone ^210^Pb (20.7 Bq/kg wm, recalculated from dry mass) was similar to or lower than values reported for wolves from two Canadian areas (36 and 165 Bq/kg wm; [30]). These differences, when compared with more northern wolf populations, are not surprising given the pronounced variation in prey species and their higher ^137^Cs activity. Moose (*Alces alces*) and reindeer (*Rangifer tarandus*) in Sweden ([29] and references cited therein) and caribou in the Northwest Territories of Canada (*Rangifer tarandus*, [30]), which dominate wolf diets in those regions, accumulate ^137^Cs to a much greater extent than the roe deer, wild boar, or domestic animals [47] that constitute the main prey of Croatian wolves [53,54]. An earlier study of large Croatian carnivores reported muscle ^137^Cs concentrations in wolves (0.23–22.2 Bq/kg wm, *n* = 7) from the 2012–2014 period comparable to those observed in this study (0.287–48.2 Bq/kg wm), with a similar mean but a higher median (17.5 *vs*. 1.62 Bq/kg wm; [28]). The same authors found that wolves had muscle ^137^Cs levels similar to brown bears, higher than golden jackals, and lower than lynx from the same area. Gjelsvik et al. [29] likewise identified Scandinavian lynx as the carnivore with the highest muscle ^137^Cs concentrations, followed by grey wolves and wolverines (*Gulo gulo*). Radiation doses calculated for large carnivores with even higher ^137^Cs levels [19,41] than quantified in this study were not expected to result in adverse health effect.

#### 4.1.2. Distribution Among Tissues

Radiocaesium distribution in the heart and lungs reported for Swedish lynx (≈50% relative to muscle) agrees with the present results for wolves (61% and 37%, respectively), whereas relative ^137^Cs levels in lynx liver (≈80%) and kidney (≈90%; [19]) were higher than those observed in Croatian wolves (58% and 59%, respectively). The distribution of ^137^Cs among organs of wild Japanese monkeys (*Macaca fuscata* [77]) was similar to that observed in this study, except for the lungs, where accumulation was higher (70% *vs*. 37% relative to muscle in wolves).

In domestic animals, such as pigs and cattle, the distribution of ^137^Cs typically followed the following pattern: muscle > kidney > heart > spleen > liver [34,69]. Biokinetic models predict the highest ^137^Cs accumulation in tissues containing the majority of the body’s intracellular K, such as muscles [86]. Exposure conditions strongly influence tissue distribution, while the residual blood content can reduce apparent tissue ^137^Cs activity [69], which helps to explain differences among studies. Correlation analysis revealed a strong association between muscle ^137^Cs and that in other tissues of wolves (Figure 1a). This finding suggests that ^137^Cs distribution is largely governed by common physiological and exposure-related processes, thereby supporting the use of muscle ^137^Cs massic activity as a proxy for overall body burden [38,43].

#### 4.1.3. Concentration Ratios

Concentration ratios are widely used to predict radionuclide massic activities in organisms based on soil activities within their habitat. Most available studies on large carnivores originate from Scandinavia, where the non-linear transfer of ^137^Cs from soil to lynx and bear tissues [19,79,84] at high soil, shrub and prey (e.g., *Rangifer* spp.) massic activities could prevent the usage of derived transfer parameters as a part of carnivorous mammals group [12]. In some species, such as wild boar, this non-linearity arises from the slow decline of ^137^Cs activity in muscle tissue despite rapid decreases in soil activity [87]. These factors underscore the importance of the present data on grey wolves, which provide rarely available transfer parameters for a species relevant to temperate forests of many European countries with comparably low environmental (i.e., soil and prey species) ^137^Cs and ^210^Pb activities.

When comparing concentration ratio (CR_whole organism-soil_) values derived from stable Pb and ^210^Pb in wolves (Table 4), differences of up to one order of magnitude were observed, as previously noted by other authors [39,44,45]. Geographical bias in radionuclide and stable analogue data or different sources has been proposed as a possible explanation for this discrepancy [40,44,45]. Literature CRs vary substantially among mammalian subgroups defined by feeding strategy and compared to values calculated for Croatian grey wolves (Table 4). Wolves, as carnivores feeding primarily on large herbivorous species, exhibit lower Cs and Pb transfer rates than their herbivorous and omnivorous prey—and lower than might be expected for an apex predator—despite earlier assumptions that top predators accumulate radionuclides more efficiently from soil than species at lower trophic levels ([39] and references therein). Small insectivorous mammals, in contrast, display notably higher transfer rates than wolves, thereby contributing to the higher overall CRs for carnivore groups, as noted in the wildlife transfer database [39]. CR values calculated in this study from stable Cd data, as well as CRs for Pb derived from ^210^Pb data (but not those derived from stable Pb data), are in good agreement with IAEA transfer values for carnivorous mammals (Table 4) and with the default value defined in the ERICA Tool for dose rate estimations in large mammals (CR for Pb = 0.0385). In contrast, CRs calculated for Ca, Cs, Cu, Mn, and Zn differ by at least one order of magnitude from literature values for carnivorous and other mammalian groups (Table 4) and from default ERICA values (CR for Cs = 2.85). In addition to variability between studies, our results highlight a clear discrepancy between soil-to-wolf transfer values for ^210^Pb and stable Pb, although tissue levels were strongly associated. Therefore, stable Pb does not appear to be a suitable analogue for calculating CR and estimating ^210^Pb transfer. Similar discrepancies between radionuclide and their stable analogue transfer values (CRs) have been reported for other pairs, such as ^90^Sr *vs*. Sr in bears [44], ^137^Cs *vs*. Cs and ^40^K *vs*. K in roe deer [45], or ^210^Pb *vs*. Pb in *Rangifer* spp. [39]. Relatively small differences in ^40^K *vs*. K CR values have been attributed to a common source of the radionuclide and stable element, whereas more pronounced deviations in ^137^Cs/Cs CR values reflect differences in origin, namely aerial deposition *vs*. parent rock sources ([45] and references cited therein).

### 4.2. Stable Elements

#### 4.2.1. Non-Essential Elements

The primary source of exposure to non-essential elements in wolves is diet, which in Croatian populations consists mainly of wild even-toed ungulates and domestic animals in proportions that vary regionally [53,54]. Some studies suggest that prey choice is a more important determinant of pollutant levels than the spatial variability of pollutants in the environment ([24] and references therein; [88]). This pattern reflects the transfer of pollutants from soil, water, and air to plants and subsequently to herbivores or omnivores, with progressive accumulation across trophic levels and ultimately elevated exposure in apex predators [17]. Wolves are opportunistic yet obligate predators ([53] and references therein); therefore, the availability of prey and the pollutant load in consumed tissues largely determine the exposure level of individual animals. An earlier study based on a larger sample of Croatian wolves (*n* = 127) suggested that both diet and geological characteristics contribute to regional differences in pollutant levels [22]. However, more pronounced diet-related differences have been documented among Alaskan wolf populations [20,24,88], where studied packs occupy geographically distinct ranges without overlap, unlike the relatively small Croatian territory. Based on conclusions from Alaskan studies, it may be hypothesized that wolves from the northern Croatian area, which feed predominantly on wild ungulates, could be more exposed to Cd and Pb through prey tissues than southern populations that rely more heavily on domestic animals [89]. However, this hypothesis could not be tested in the present study because of the small sample size (*n* = 10) and the lack of information on the home ranges of individual wolves, which would be required to assign animals to specific regions beyond the currently available data on the location of death.

Global spatial variation in tissue levels of non-essential elements reported for grey wolves from temperate and sub-Arctic regions of Europe and North America is summarized in Table A2. Sub-Arctic regions, typically located north of approximately the 50th parallel, are characterized by climate-driven vegetation (e.g., lichens and mosses) and herbivorous fauna (e.g., reindeer/caribou and moose), which tend to exhibit higher trace element levels [90] than ungulates from temperate habitats [91]. Hoffman et al. [92] reported approximately fivefold higher renal Cd in wolves from North American Arctic and sub-Arctic regions compared with wolves from more southern latitudes. Accordingly, the generally higher As, Cd, and Hg levels observed in wolves from Yukon and Alaska compared with Croatian wolves (Table A2) were expected. However, substantial variability in the levels of major pollutants (Cd, Hg, and Pb) has also been documented within Europe (e.g., Iberian wolf population [25]), independent of latitude or feeding strategy, as noted by Lazarus et al. [22]. This suggests that regional geochemistry and long-range atmospheric transport are also important drivers of pollutant exposure. Overall, concentrations of As, Ba, Cd, Cs, Hg, Pb, Sr, Tl, and U in Croatian wolves are within or below the ranges reported for other wolf populations in Europe and North America.

In addition, levels of non-essential elements may be influenced by biological factors such as sex, age, body condition, overall health status, social rank within the pack, or dispersal behavior [22,24]. However, none of the factors examined here (age, sex, and BCI) had a significant effect on the renal Cd and femoral Pb. Conflicting results regarding the effects of age and sex on non-essential elements in different wolf tissues worldwide have been discussed in detail elsewhere [22].

#### 4.2.2. Essential Elements

Because of their important physiological roles, organisms maintain relatively stable concentrations of essential elements in blood and tissues through homeostatic regulation. Nevertheless, certain elements, such as Fe, Cu, Se, and Zn, may accumulate in organs at levels capable of causing toxic effects, while deficiencies of these elements can also adversely affect animal health. Baseline data on normal ranges of essential elements in wolves are scarce in the literature. Therefore, we compared the concentrations measured in this study with those reported previously for a larger wolf dataset from the same region [22] and found no substantial differences (Table A2). When evaluated against reference ranges established for domestic dogs [75], some individuals in this study may have experienced Fe and Mn deficiency, whereas others showed elevated levels of Fe, Mn, and V (Table 5). However, these interpretations should be treated with caution because physiological reference ranges derived from domestic dogs may not accurately reflect the normal elemental status of wolves.

### 4.3. Limitations and Future Work

A key challenge in studies involving apex predators such as wolves is obtaining an adequate sample size due to stringent legal protection and ethical considerations. These often limit opportunities for invasive sampling to incidental or post-mortem collections (e.g., roadkill, culling under management plans, or natural deaths). Future research would benefit from cross-border collaborative frameworks integrating pollutants, pathogens, and the ecology of sentinel species, including ethically compliant long-term monitoring programs conducted in partnership with wildlife management authorities, to help to mitigate current limitations while adhering to conservation priorities outlined in the European Commission’s The European Green Deal [93].

## 5. Conclusions

To our knowledge, this is the first study to report ^137^Cs and ^40^K massic activities, together with stable element levels, in the heart, spleen, and lungs of grey wolves. Combined with data from the muscle, liver, kidney, and femoral bone of wolves, this represents one of the most comprehensive assessments of inorganic pollutant exposure in this protected species, with relevance for the conservation and management of European wolf populations inhabiting temperate habitats. The distribution of ^137^Cs was consistent with patterns reported for other mammals and showed strong correlation within tissues, while, for Cd and Pb, associations were generally weaker. Constrained by a low number of individuals and high ^137^Cs massic activity in one wolf, correlations between radionuclides and their stable analogues in wolf tissues highlight the uncertainty in using stable element data in radiological risk assessment. In addition, due to differences in source terms and the geogenic origin of radionuclides and their stable counterparts in soils, site-specific transfer parameters (CR_whole organism-soil_) derived from stable element data should be applied with caution when radionuclide data are lacking. Although based on a limited sample size, our results suggest that grey wolves, as apex terrestrial predators, are not currently at risk of adverse health effects from exposure to the most relevant inorganic anthropogenic pollutants.

## Figures and Tables

**Table 2:** Radionuclide massic activity and their stable element analogues levels expressed on a dry tissue mass (AM ± SD, GM, median, range) in tissues of ten grey wolves (*Canis lupus*) sampled during the 2014–2018 period in Croatia ^a^.

Tissue		^137^Cs (Bq/kg)	Cs (μg/kg)	^40^K (Bq/kg)	K (mg/kg)	^210^Pb (Bq/kg)	Pb (μg/kg)
Muscle	AM ± SD GM, median Min–max	40.5 ± 70.0 12.0, 6.49 1.15–193	467 ± 850 216, 172 67.6–2854	419 ± 30 418, 432 354–441	12,407 ± 1547 12,318, 12,285 9582–14,849		8.09 ± 6.35 5.34, 7.28 0.927–19.0
Heart	AM ± SD GM, median Min–max	22.4 ± 37.7 7.09, 3.39 0.688–105	255 ± 426 128, 113 36.8–1446	342 ± 57 337, 351 241–398	10,102 ± 1297 10,017, 10,057 7078–11,582		12.4 ± 9.4 9.00, 10.9 2.34–30.5
Spleen	AM ± SD GM, median Min–max	24.2 ± 45.2 5.99, 3.60 0.527–125	289 ± 537 128, 112 35.4–1801	303 ± 81 293, 323 171–402	9905 ± 1777 9730, 10,280 5758–11,910		104 ± 102 68.6, 81.5 10.2–361
Lungs	AM ± SD GM, median Min–max	16.4 ± 31.9 4.32, 2.34 0.564–88.0	120 ± 227 55.0, 45.0 17.4–763	194 ± 61 185, 191 119–284	4129 ± 1204 3959, 4309 2453–5732		98.3 ± 128 58.9, 39.9 24.2–434
Bone	AM ± SD GM, median Min–max	6.68 ± 12.32 1.56, 0.558 0.377–28.6	39.4 ± 80.1 15.2, 11.6 3.46–265	55.5 ± 18.7 53.4, 52.3 41.1–87.5	1470 ± 451 1408, 1369 854–2253	25.9 ± 21.9 20.8, 16.4 11.8–63.9	2198 ± 1164 1844, 2089 402–4301
Liver	AM ± SD GM, median Min–max	20.9 ± 35.6 6.14, 3.90 0.526–99.5	222 ± 401 106, 86.2 27.6–1352	229 ± 38 226, 225 172–285	6921 ± 959 6863, 6747 5796–8574		572 ± 487 387, 438 108–1455
Kidney	AM ± SD GM, median Min–max	24.3 ± 43.3 6.69, 3.71 0.373–120	285 ± 531 132, 119 42.9–1783	250 ± 61 243, 251 142–330	8235 ± 1166 8166, 7758 7003–10,689		629 ± 610 418, 498 102–2105

^a 137^Cs and ^40^K measured in soft tissues of seven wolves; ^210^Pb measured in femoral bone of five wolves; AM—arithmetic mean, SD—standard deviation, GM—geometric mean.

**Table 3:** Correlation analysis results between radionuclides and their stable analogues within the tissues of grey wolves (*Canis lupus*) sampled during the 2014–2018 period in Croatia ^a^.

Tissue	Radionuclide *vs*. Stable Element	Bayesian Mean (95% HDI)	Radionuclide *vs*. Stable Element	Bayesian Mean (95% HDI)
muscle	^137^Cs & Cs	0.72 (0.11, 1.00)	^40^K & K	0.23 (−0.45, 0.84)
heart		0.90 (0.61, 1.00)		0.68 (0.16, 0.98)
spleen		0.83 (0.42, 1.00)		0.59 (0.02, 0.99)
lungs		0.70 (0.07, 1.00)		0.68 (0.16, 0.99)
bone		0.85 (0.26, 1.00)		0.57 (−0.14, 0.99)
liver		0.89 (0.59, 1.00)		0.83 (0.51, 0.99)
kidney		0.82 (0.39, 1.00)		0.70 (0.23, 0.99)
bone	^210^Pb & Pb	0.65 (−0.02, 0.99)		

^a^ *n* = 7, except for ^210^Pb & Pb in femoral bone, where *n* = 5; HDI, highest density interval.

**Table 4:** Concentration ratio values (CR_wo/soil_) for terrestrial ecosystem species and groups (AVG ± SD, GM, range) calculated as fresh weight whole organism concentration/dry weight soil concentration of respective radionuclide or stable element.

Element	Wolf ^a^	Carnivorous Mammal ^b^	Herbivorous Mammal ^c^	Omnivorous Mammal ^d^	Mammal ^e^
Ca	0.0054 ± 0.0038 0.0047 0.0031–0.016				8.5 ± 15 4.3 0.022–47
Cd	1.01 ± 0.70 0.791 0.200–2.18	1.3 ± 2.9 0.54 0.085–21	6.8 ± 0.89 6.7		
Cu	0.059 ± 0.018 0.056 0.035–0.095				0.31 ± 0.15 0.28 0.073–0.43
Cs	*^137^Cs* 0.089 ± 0.152 0.026 0.003–0.419	0.54 ± 1.9 0.14 0.0028–23	3.8 ± 8.4 1.8 0.01–140	3.3 ± 6 1.0 0.017–36	
Mn	0.00052 ± 0.00011 0.00051 0.00040–0.00068	0.0025 ± 0.00082 0.0024 0.0019–0.0036			
Pb	*^210^Pb* 0.035 ± 0.029 0.028 0.016–0.086	0.047 ± 0.028 0.04 0.0088–0.077	0.02 ± 0.027 0.012 0.0019–0.2	0.012 ± 0.063 0.0022 0.00027–0.039	
	*Pb stable* 0.0064 ± 0.0034 0.0054 0.0012–0.0126				
Zn	0.438 ± 0.454 0.344 0.161–0.385				3.0 ± 2.2 2.4 0.22–7.8

^a^ N = 7 for Cs radionuclide, *n* = 5 for Pb radionuclide, *n* = 10 for stable Ca, Cd, Cu, Mn, Pb, and Zn; this study. ^b^ N = 395 for Cd, *n* = 231 for Cs, *n* = 4 for Mn (shrews), *n* = 368 for Pb; [38,39]. ^c^ N = 1879 for Cs, *n* = 92 for Pb, *n* = 20 for Cd; [38,39]. ^d^ N = 333 for Cs, *n* = 51 for Pb; [38,39]. ^e^ N = 17 for Ca, Cu, and Zn; [70].

**Table 5:** Threshold values of toxic effects for non-essential elements or adequate values listed for essential elements (mg/kg dry mass).

Element (mg/kg dm)	Tissue	Wolf Levels (Range, This Study)	Threshold Level of Toxic Effects	Source
As	Liver	0.0030–0.0327	30.3 ^a^	[75]
	Kidney	0.0098–0.0827	40 ^a^	[75]
Ca	Liver	102–440	100–757 ^a^ adequate	[75]
	Kidney	267–558	2000 ^a^ high	[75]
Cd	Liver	0.068–0.859	3.03–21.2 ^a^ high	[75]
	Kidney	0.309–4.50	120 ^a^	[71]
			400 ^a^	[72]
			800 ^a^	[75]
Cu	Liver	9.01–116	1212 ^a^	[75]
	Kidney	10.2–42.9	80 ^a^	[75]
Fe	Liver	176–1223	424 ^a^ adequate	[75]
	Kidney	98.1–1295	560 ^a^ adequate	[75]
Hg	Liver	0.0481–0.785	91 ^a^	[73]
	Kidney	0.0412–2.31	120 ^a^	[73]
Mn	Liver	4.82–19.0	9.09–15.1 ^a^ adequate	[75]
	Kidney	3.45–6.61	4.8–7.2 ^a^ adequate	[75]
Pb	Liver	0.108–1.45	25–30	[74]
	Kidney	0.102–2.10	15	[74]
	Bone	0.402–4.30	25	[74]
Se	Liver	1.20–2.66	20	[76]
	Kidney	3.26–6.20	4–6 ^a^ adequate	[75]
Tl	Liver	0.0014–0.0105	23.0 ^a^	[75]
	Kidney	0.0034–0.0335	136 ^a^	[75]
U	Kidney	0.0013–0.013	4–9.2 ^a^	[75]
V	Liver	0.066–0.673	0.09–0.182 ^a^ adequate	[75]
	Kidney	0.088–0.438	0.12–0.2 ^a^ adequate	[75]
Zn	Liver	56.8–606	1118 ^a^	[75]
	Kidney	75.7–229	1180 ^a^	[75]

^a^ Recalculated to dry mass using f = 3.7 for muscle, f = 3.03 for liver and f = 4 for kidney [22].

## Data Availability

The data presented in this study are available on request from the corresponding author. The data are not publicly available due to privacy issues.

## Appendix A

### Appendix A.1

**Table A1:** Stable element levels expressed on a dry tissue mass (AM ± SD, GM, median, range) in tissues of ten grey wolves (*Canis lupus*) sampled during the 2014–2018 period in Croatia ^a^.

Element		Tissue						
		Muscle	Heart	Spleen	Lungs	Bone	Liver	Kidney
As (μg/kg)	AM ± SD GM, med Min–max	20.6 ± 13.5 17.4, 16.7 7.99–49.6	9.62 ± 6.30 8.27, 6.64 4.51–24.6	8.72 ± 4.20 8.01, 6.86 4.85–18.7	4.07 ± 2.56 3.36, 3.27 <1.75–8.46	104 ± 14 103, 99.3 88.5–129	12.0 ± 9.5 9.23, 9.13 3.05–32.7	22.7 ± 22.4 17.4, 13.4 9.79–82.7
Ba (μg/kg)	AM ± SD GM, med Min–max	21.5 ± 16.2 17.3, 17.2 7.37–56.5	15.1 ± 8.3 13.3, 11.4 7.67–30.7	76.0 ± 68.8 54.6, 42.5 14.9–206	67.9 ± 47.6 54.5, 42.3 22.0–158	28,277 ± 18,948 24,240, 22,151 11,285–71,380	74.7 ± 125 32.4, 22.1 5.81–414	87.4 ± 77.1 63.8, 50.2 24.2–254
Ca (mg/kg)	AM ± SD GM, med Min–max	206 ± 145 179, 146 117–596	165 ± 49 158, 139 105–247	258 ± 47 254, 258 182–344	292 ± 75 283, 275 176–407	305,839 ± 16,697 305,434, 302,275 284,207–336,512	195 ± 96 179, 181 102–440	343 ± 89 334, 307 267–558
Cd (μg/kg)	AM ± SD GM, med Min–max	3.42 ± 2.40 2.69, 2.49 0.681–7.42	9.00 ± 6.17 6.82, 8.42 1.21–21.1	49.5 ± 29.9 39.3, 51.8 9.27–104	19.1 ± 13.6 13.6, 19.7 2.08–42.4	0.938 ± 0.993 0.494, 0.542 <0.180–3.12	301 ± 273 213, 207 68.2–859	1180 ± 1332 797, 601 309–4503
Co (μg/kg)	AM ± SD GM, med Min–max	15.4 ± 9.0 13.4, 12.3 6.68–33.7	112 ± 138 80.3, 67.5 35.9–501	44.4 ± 30.2 37.2, 37.6 20.3–106	32.6 ± 27.5 25.3, 22.2 9.46–98.4	349 ± 19 348, 350 319–384	62.4 ± 33.4 56.9, 53.0 29.7–150	69.8 ± 35.4 62.9, 55.7 33.2–145
Cu (mg/kg)	AM ± SD GM, med Min–max	7.09 ± 2.18 6.80, 7.18 4.28–11.5	20.6 ± 3.1 20.3, 21.1 13.5–24.9	3.47 ± 1.33 3.30, 3.09 2.16–6.94	2.48 ± 2.08 2.10, 1.85 1.38–8.32	1.68 ± 2.08 0.751, 0.865 0.063–6.67	47.9 ± 34.1 36.4, 38.5 9.01–116	20.1 ± 8.9 18.8, 17.9 10.2–42.9
Fe (mg/kg)	AM ± SD GM, med Min–max	184 ± 38 180, 189 114–234	312 ± 97 302, 292 230–571	1078 ± 354 1023, 1151 582–1693	895 ± 444 784, 880 283–1670	6.31 ± 13.8 <3.05–45.5	602 ± 311 527, 575 176–1223	514 ± 359 411, 383 98.1–1295
Hg (μg/kg)	AM ± SD GM, med Min–max	71.5 ± 94.7 33.9, 36.5 <5.65–319	66.5 ± 85.0 37.0, 35.2 7.61–284	60.7 ± 54.2 43.8, 43.9 9.57–193	20.6 ± 27.8 11.6, 9.83 <5.65–95.5	67.1 ± 89.9 38.0, 22.6 20.7–240	197 ± 231 127, 101 48.1–785	754 ± 780 425, 512 41.2–2309
Mn (mg/kg)	AM ± SD GM, med Min–max	0.883 ± 0.232 0.857, 0.802 0.625–1.23	1.84 ± 0.53 1.79, 1.76 1.32–3.09	1.44 ± 0.44 1.38, 1.34 0.924–2.46	0.503 ± 0.414 0.422, 0.418 0.175–1.66	0.598 ± 0.122 0.587, 0.594 0.462–0.776	13.4 ± 4.8 12.4, 13.9 4.82–19.0	5.42 ± 1.11 5.30, 5.57 3.45–6.61
Mo (μg/kg)	AM ± SD GM, med Min–max	13.8 ± 6.9 12.3, 12.3 5.32–27.7	46.5 ± 15.5 44.2, 41.8 29.1–68.3	91.8 ± 114 88.7, 88.8 50.8–128	65.0 ± 24.3 61.0, 59.8 32.8–108	<1.95	985 ± 361 925, 936 487–1561	791 ± 188 768, 846 437–1038
Se (mg/kg)	AM ± SD GM, med Min–max	0.428 ± 0.234 0.394, 0.356 0.288–1.08	0.956 ± 0.315 0.918, 0.808 0.718–1.60	1.27 ± 0.25 1.25, 1.22 1.01–1.72	0.810 ± 0.151 0.798, 0.817 0.552–1.14	0.043 ± 0.036 0.032, 0.036 0.0056–0.116	1.60 ± 0.41 1.56, 1.51 1.20–2.66	4.99 ± 0.91 4.91, 5.29 3.26–6.20
Sr (μg/kg)	AM ± SD GM, med Min–max	52.9 ± 42.9 41.0, 37.5 16.0–155	55.6 ± 44.2 41.5, 38.6 13.1–133	103 ± 79 84.4, 83.3 31.8–312	93.2 ± 67.6 75.7, 59.0 30.0–211	69,905 ± 44,822 57,887, 48,067 27,911–134,747	82.2 ± 107.4 54.6, 52.6 17.0–382	115 ± 85 90.8, 83.9 34.6–254
Tl (μg/kg)	AM ± SD GM, med Min–max	4.25 ± 1.80 3.77, 4.64 1.04–6.46	7.92 ± 3.95 6.97, 7.37 2.43–13.8	6.30 ± 2.95 5.64, 5.70 2.20–12.0	2.51 ± 1.32 2.18, 2.16 0.597–5.14	2.77 ± 1.17 2.60, 2.40 1.58–5.57	5.62 ± 3.36 4.63, 4.66 1.38–10.5	20.9 ± 9.5 17.9, 22.5 3.36–33.5
U (μg/kg)	AM ± SD GM, med Min–max	<0.026–0.089	<0.026–0.0313	0.105 ± 0.060 0.093, 0.080 0.044–0.247	0.189 ± 0.185 0.140, 0.098 0.051–0.664	2.58 ± 1.72 2.17, 2.43 0.740–6.84	0.229 ± 0.137 0.198, 0.193 0.092–0.500	5.21 ± 3.90 4.02, 3.96 1.32–13.3
V (μg/kg)	AM ± SD GM, med Min–max	6.76 ± 5.74 5.30, 4.94 2.51–20.0	18.7 ± 15.4 14.8, 12.8 7.21–52.7	95.1 ± 82.7 74.8, 62.6 33.4–303	17.5 ± 12.0 14.9, 11.7 7.26–44.9	296 ± 203 252, 235 97.0–808	269 ± 219 206, 202 66.0–673	198 ± 109 175, 157 88.4–438
Zn (mg/kg)	AM ± SD GM, med Min–max	189 ± 61 179, 198 104–276	104 ± 9 104, 105 87.3–121	124 ± 24 122, 129 82.1–169	36.6 ± 9.6 35.3, 36.2 19.6–49.3	104 ± 11 103, 105 88.9–123	155 ± 160 121, 115 56.8–606	112 ± 43 107, 101 75.7–229

^a^ AM—arithmetic mean, SD—standard deviation, GM—geometric mean, med—median.

### Appendix A.2

**Table A2:** Levels of stable elements (median, expressed on a dry mass) in tissues of grey wolves from the literature grouped by European and North American countries, and within continents from southern to more northern latitudes.

Element	Country, Period	*n*	Tissue				Source
			Muscle	Bone	Liver	Kidney	
As (μg/kg)	Croatia (2014–2018)	10	16.7	99.3	9.13	13.4	This study
	Croatia (2008–2013) ^a^	125	12.2		7.64	13.7	[22]
	Croatia (2009–2010) ^a,b^	4	29.6		24.2	36	[67]
	Norway (1993–2006) ^c^	39	46		15	12	[26]
	Yukon, Canada (1993–1994) ^c^	21			203	333	[27]
Ba (μg/kg)	Croatia (2014–2018)	10	17.2	22,151	22.1	50.2	This study
	Serbia (2011–2015) ^a,c^	28			879		[23]
	Norway (1993–2006)	39	70		230	520	[26]
	Minnesota, USA (2012–2013) ^a,c^	147			788		[21]
Ca (mg/kg)	Croatia (2014–2018)	10	146	302,275	181	307	This study
	Croatia (2008–2013)	125	160		205	302	[22]
	Minnesota, USA (2012–2013) ^a,c^	147			170		[21]
Cd (μg/kg)	Croatia (2014–2018)	10	2.49	0.542	207	601	This study
	Croatia (2008–2013)	125	1.01		161	676	[22]
	Croatia (2009–2010) ^a,b^	4	37		257	996	[67]
	Spain (2008–2011) ^c^	33			528	2692	[25]
	Serbia (2011–2015) ^a,c^	28			273		[23]
	Norway (1993–2006) ^c^	39	4		440	1050	[26]
	Minnesota, USA (2012–2013) ^a,c^	147			788		[21]
	Idaho, USA (2005–2008) ^a,b^	28				1120	[92]
	Montana, USA (2005–2008) ^a,b^	32				1840	[92]
	NWT, Canada (2005–2008) ^a,b^	42				2800	[92]
	Alaska, USA (2005–2008) ^a,b^	13				6200	[92]
	Yukon, Canada (1993–1994) ^c^	21			1420	5930	[27]
Co (μg/kg)	Croatia (2014–2018)	10	12.3	350	53.0	55.7	This study
	Croatia (2008–2013)	125	9.92		67.6	91.2	[22]
	Serbia (2011–2015) ^a,c^	28			91		[23]
	Norway (1993–2006) ^c^	39	10		43	42	[26]
Cs (μg/kg)	Croatia (2014–2018)	10	172	11.6	86.2	119	This study
	Norway (1993–2006) ^c^	39	1270		650	820	[26]
Cu (mg/kg)	Croatia (2014–2018)	10	7.18	0.865	38.5	17.9	This study
	Croatia (2008–2013)	125	8.21		40.9	23.2	[22]
	Croatia (2009–2010) ^a,b^	4	2.74		19.5	13.3	[67]
	Serbia (2011–2015) ^a,c^	28			16.7		[23]
	Norway (1993–2006) ^c^	39	9.2		72.0	24.7	[26]
	Minnesota, USA (2012–2013) ^a,c^	147			34.6		[21]
	Idaho, USA (2005–2008) ^a,b^	28				23.9	[92]
	Montana, USA (2005–2008) ^a,b^	32				22.2	[92]
	NWT, Canada (2005–2008) ^a,b^	42				28.0	[92]
	Alaska, USA (2005–2008) ^a,b^	13				26.8	[92]
	Yukon, Canada (1993–1994) ^c^	21			48.78	20.8	[27]
Fe (mg/kg)	Croatia (2014–2018)	10	189	<3.05	575	383	This study
	Croatia (2008–2013)	125	124		457	345	[22]
	Serbia (2011–2015) ^a,c^	28			467		[23]
	Minnesota, USA (2012–2013) ^a,c^	147			627		[21]
	Idaho, USA (2005–2008) ^a,b^	28				384	[92]
	Montana, USA (2005–2008) ^a,b^	32				404	[92]
	NWT, Canada (2005–2008) ^a,b^	42				402	[92]
	Alaska, USA (2005–2008) ^a,b^	13				619	[92]
Hg (μg/kg)	Croatia (2014–2018)	10	36.5	22.6	101	512	This study
	Croatia (2008–2013)	125	16.3		41.8	142	[22]
	Croatia (2009–2010) ^a,b^	4	22.2		51.5	32	[67]
	Serbia (2011–2015) ^a,c^	28			454		[23]
	Slovenia (1991–1997) ^a,c^	2	32.9		147	411	[68]
	Norway (1993–2006) ^c^	39	46		220	570	[26]
	Alaska, USA (2006–2009) ^a^	162	46.2		105	381	[24]
	Alaska, USA ^a,d^	7–34	74–851		121–194,829	560–1160	[20]
	Yukon, Canada (1993–1994) ^c^	21			543	1110	[27]
K (mg/kg)	Croatia (2014–2018)	10	12,285	1369	6747	7758	This study
	Croatia (2008–2013)	125	13,225		7580	9764	[22]
	Minnesota, USA (2012–2013) ^a,c^	147			7602		[21]
Mn (mg/kg)	Croatia (2014–2018)	10	0.802	0.594	13.9	5.57	This study
	Croatia (2008–2013)	125	0.850		10.2	5.84	[22]
	Serbia (2011–2015) ^a,c^	28			11.2		[23]
	Norway (1993–2006) ^c^	39	0.97		13.0	5.1	[26]
	Minnesota, USA (2012–2013) ^a,c^	147			8.82		[21]
Mo (μg/kg)	Croatia (2014–2018)	10	12.3	<1.95	936	846	This study
	Croatia (2008–2013)	125	13.3		1091	886	[22]
	Serbia (2011–2015) ^a,c^	28			2394		[23]
	Norway (1993–2006) ^c^	39	138		790	510	[26]
	Minnesota, USA (2012–2013) ^a,c^	147			939		[21]
Pb (μg/kg)	Croatia (2014–2018)	10	7.28	2089	438	498	This study
	Croatia (2008–2013)	125	5.85		367	439	[22]
	Croatia (2009–2010) ^a,b^	4	77.7		206	128	[67]
	Serbia (2011–2015) ^a,c^	28			200		[23]
	Spain (2008–2011) ^c^	33			4108	31	[25]
	Germany (2017–2021)	32		990			[94]
	Norway (1993–2006) ^c^	39	740		390	190	[26]
	Minnesota, USA (2012–2013) ^a,c^	147			3212		[21]
	Yukon, Canada (1993–1994) ^c^	21		742	243	652	[27]
Se (mg/kg)	Croatia (2014–2018)	10	0.356	0.036	1.51	5.29	This study
	Croatia (2008–2013)	125	0.325		1.25	4.58	[22]
	Norway (1993–2006) ^c^	39	0.31		1.50	4.03	[26]
	Yukon, Canada (1993–1994) ^c^	21			3.38	5.23	[27]
Sr (μg/kg)	Croatia (2014–2018)	10	37.5	48,067	52.6	83.9	This study
	Serbia (2011–2015) ^a,c^	28			1485		[23]
	Norway (1993–2006) ^c^	39	210		180	260	[26]
Tl (μg/kg)	Croatia (2014–2018)	10	4.64	2.40	4.66	22.5	This study
	Croatia (2008–2013)	125	3.53		5.03	14.2	[22]
	Norway (1993–2006) ^c^	39	3.1		3.9	28.4	[26]
U (μg/kg)	Croatia (2014–2018)	10	<0.026	2.43	0.193	3.96	This study
	Croatia (2008–2013)	125	0.063		0.214	2.80	[22]
V (μg/kg)	Croatia (2014–2018)	10	4.94	235	202	157	This study
	Norway (1993–2006) ^c^	39	28		38	21	[26]
Zn (mg/kg)	Croatia (2014–2018)	10	198	105	115	101	This study
	Croatia (2008–2013)	125	134		97.9	94.7	[22]
	Serbia (2011–2015) ^a,c^	28			73.0		[23]
	Spain (2008–2011) ^c^	33			77.9	25.8	[25]
	Norway (1993–2006) ^c^	39	136		148	99	[26]
	Minnesota, USA (2012–2013) ^a,c^	147			102		[21]
	Idaho, USA (2005–2008) ^a,b^	28				102	[92]
	Montana, USA (2005–2008) ^a,b^	32				112	[92]
	NWT, Canada (2005–2008) ^a,b^	42				111	[92]
	Alaska, USA (2005–2008) ^a,b^	13				150	[92]
	Yukon, Canada (1993–1994) ^c^				104	86.3	[27]

^a^ Recalculated to dry mass using f = 3.7 for muscle, f = 3.03 for liver and f = 4 for kidney [22]; ^b^ geometric mean; ^c^ arithmetic mean; ^d^ range.
